# Improving Safety Among Pregnant Women Reporting Domestic Violence in Nepal—A Pilot Study

**DOI:** 10.3390/ijerph17072268

**Published:** 2020-03-27

**Authors:** Poonam Rishal, Kunta Devi Pun, Berit Schei, Buna Bhandari, Sunil Kumar Joshi, Katarina Swahnberg, Jennifer Jean Infanti, Mirjam Lukasse, the ADVANCE study group

**Affiliations:** 1 Department of Public Health and Nursing, Faculty of Medicine and Health Sciences, NTNU, Norwegian University of Science and Technology, Postbox 8905, 7491 Trondheim, Norway; poonam.rishal1@gmail.com (P.R.); knt_pun@yahoo.com (K.D.P.); Berit.Schei@ntnu.no (B.S.); jennifer.infanti@ntnu.no (J.J.I.); 2 Department of Community Medicine, Kathmandu Medical College and Teaching Hospital, PO Box 21266, Sinamangal, Kathmandu 44600, Nepal; drsunilkumarjoshi@gmail.com; 3 Kathmandu University School of Medical Sciences, Kathmandu University and Dhulikhel Hospital, Dhulikhel 45200, Bagmati, Nepal; 4 Department of Obstetrics and Gynecology, St. Olavs University Hospital, Postbox 3250, Sluppen, 7006 Trondheim, Norway; 5 Department of Community Medicine and Public Health, Institute of Medicine, Tribhuvan University, Kathmandu 44600, Nepal; buna.bhandari@gmail.com; 6 School of Public Health and Community Medicine, UNSW Medicine, University of New South Wales, Sydney, NSW 2052, Australia; 7 Department of Health and Caring Sciences, Faculty of Health and Life Sciences, Linnæus University, 391 82 Kalmar, Sweden; katarina.swahnberg@lnu.se; 8 Department of Nursing and Health Promotion, Faculty of Health Sciences, Oslo Metropolitan University, Postbox 4, St. Olavs Plass, 0130 Oslo, Norway; 9 Department of Health and Social Sciences, University College of Southeast Norway, Postbox 235, 3603 Kongsberg, Campus Vestfold, 3184 Borre, Norway

**Keywords:** domestic violence, pregnancy, safety measures, safety behaviors, intervention, antenatal care, Nepal

## Abstract

*Introduction:* Domestic violence (DV) during pregnancy is associated with poor health outcomes for both the mother and newborn, and sometimes death. In a low-income country like Nepal, women have few options to leave abusive situations. Therefore, there is a need for interventions to improve their safety. The aim of our study was to explore the use of safety measures before and after an educational intervention among women who have reported DV during pregnancy. *Materials and methods*: Of 1010 pregnant women screened consecutively for DV using the Abuse Assessment Screen (AAS) during routine antenatal care, 181 women reported domestic violence. All 1010 participating pregnant women were taught 15 safety measures using a locally developed flipchart. We obtained contact with 80 of the 181 eligible women postpartum, of whom 62 completed the follow-up assessment. We explored and described the use of safety measures at baseline and follow-up, using a standardized instrument called the Safety Behavior Checklist. *Results:* At follow-up, less than half of the women (*n* = 30, or 48.3%) reported any form of DV. Of the women who reported DV at follow-up, significantly more reported the experience of both violence and fear at baseline (21.9%, *p* = 0.01) compared with the women who did not report DV at follow-up (3.3%, *p* = 0.01). Women reporting DV at baseline and follow-up used more safety measures at baseline (56) and follow-up (80) compared with women reporting DV at baseline only (36 and 46). Women reporting DV at baseline and follow-up used more safety measures for the first time at follow-up, 57 new measures compared with the 28 new measures used by women reporting DV at baseline only. *Conclusions*: The use of a flipchart teaching session on safety measures within antenatal care may increase the number of safety measures women use to protect themselves during pregnancy and decrease the risks of adverse health effects of DV.

## 1. Introduction

Domestic violence is defined as “any form of physical, mental, sexual and emotional harm, including acts of reprimand or emotional harm, perpetrated by one person on another with whom he or she has a family relationship” by the Ministry of Law and Justice in Nepal [1]. Globally, 30% of all women who have been in a relationship have experienced intimate partner violence, with certain regions like South-East Asia, which includes Nepal, reaching 38% [2]. In Nepal, traditionally, women relocate to their husband’s home after marriage [3], where they usually have little power. As a result, they are vulnerable to various forms of violence [4]. Additionally, living with the husband’s family can create fear, because of the power imbalances. Living in fear of someone within the family is the result of having experienced harm and/or perceived threat of harm and can thus be considered emotional abuse or a proxy for experiencing emotional violence [5].

According to the World Health Organization’s (WHO’s) multi-country study on women’s health and domestic violence, between 1% and 28% of women are subjected to physical violence during pregnancy [6]. Domestic violence (DV) during pregnancy is of particular concern because of the detrimental pregnancy outcomes associated with it, including maternal and fetal death [2]. DV during pregnancy has been associated with increased risks of unplanned pregnancy, delayed entry to prenatal care, early trimester vaginal bleeding, miscarriage, preterm birth, low birth weight, infants born small for gestational age, and postpartum depression [7,8,9,10]. 

Globally, also in Nepal, most pregnant women attend antenatal care (ANC) [11]. This offers women relationships with one or several health care providers. These relationships can provide the trust women require to disclose or report DV. ANC includes providing emotional support and medical care, education to promote safety, and appropriate referring [12,13]. Thus, ANC services hold significant potential as settings for identifying and assisting women who experience DV during pregnancy [13,14]. 

Safety planning interventions teach women who experience DV to thoroughly assess the possible threats they face and apply the most suitable measures to protect themselves [15]. Several studies have shown that women who are living in abusive relationships adopt a variety of behaviors to promote their safety as a means of coping with DV [16,17]. In Texas and Virginia (USA), a study among physically and sexually abused pregnant women found a significant increase in the use of safety measures among women being taught these, compared with women receiving care without this teaching [16]. Similarly, a pilot study among pregnant women who attended ANC in Lima, Peru reported an increase in the use of safety measures among women in the intervention group who received counseling, education, and advice on safety compared with standard care [18]. A Cochrane systematic review of 10 randomized trials assessing interventions in ANC settings suggested that counseling and education on safety-promoting measures and referral cards can reduce DV and prevent adverse outcomes by ensuring women’s safety [13].

The available evidence on ANC interventions to reduce DV and adverse outcomes among pregnant women is primarily from studies conducted in high-income countries [13]. At a policy level, Nepal has recognized DV as a violation of human rights as well as a public health issue. Despite this, ANC services have not been formally prioritized as an entry point for preventing or reducing DV [19]. For most women, regardless of context, leaving a violent relationship is difficult for several reasons: fear of being alone, shame, guilt, labelling and stigma, economic dependency, normalization of the abuse, and fear of more abuse are among factors mentioned [3,20,21]. In a rigid patriarchal society like Nepal, where marriage is regarded as a spiritual and social obligation, it is difficult for most women to leave an abusive relationship [3,22,23,24]. Women who leave face considerable economic and social challenges [23,24]. They fear breaking social traditional and socio-cultural values, fear further violence, fear marginalization and lack of justice [23,24]. Therefore, formal interventions that can mitigate the potential harms of DV to women’s physical and mental health are particularly relevant. 

The aim of our study was to explore the use of safety measures practiced before and after an educational intervention among women who have reported DV during pregnancy. 

## 2. Material and Methods

This quantitative pilot follow-up study included women disclosing domestic violence while participating in a prospective cohort study [25]. In our prospective cohort study, auxiliary nurse midwives (ANMs) at the ANC outpatient department (OPD) of Kathmandu Medical College and Teaching Hospital (KMC) consecutively assessed women attending routine care for eligibility. Women were eligible for inclusion if they were 18 years or more of age, had a pregnancy gestation between 12 and 28 weeks, understood Nepali, and could join the research assistant in a private room on their own. Eligible women were invited to participate by the research assistant. After obtaining consent, women were asked to complete a questionnaire electronically. The questionnaire included questions to screen for DV [25]. After completing the questionnaire, all participants were informed of 15 possible safety measures, by the research assistant, irrespective of whether they reported DV or not. The assistants performing the screening and teaching had been educated in safety, confidentiality, and referral procedures for the women, if required. All participants in the prospective cohort study received an information leaflet on signs and symptoms of complications during pregnancy. They could show this leaflet to their partner and family if asked about the study, without having to disclose that the study was about DV. All women were given a referral card to a safe house. Only women reporting DV in the prospective cohort study were eligible for inclusion in the pilot follow-up study, which is reported in this paper. Inclusion in the pilot follow-up study was planned to take place approximately 6 months postpartum. Recruitment to the prospective cohort study took place from August 2014 to November 2016. Recruitment was stopped temporarily because of two devastating earthquakes in Nepal on 25 April, 2015 and 12 May, 2015, respectively. The earthquakes affected most of the areas from where the women were recruited. Follow-up study interviews were done between 20 April 2016 and 26 July 2016.

### 2.1. Ethics Approval and Consent to Participate 

The study was approved by the Regional Committee for Medical and Health Research Ethics in Norway (2014/146/REK Sør-Øst C) and the Nepal Health Research Council (NHRC) (Reg.no.08/2014). At recruitment into the study during pregnancy, all women were informed and gave written consent for the follow-up study. No women were under the age of 18. Also at recruitment, all women, regardless of their report of DV, were given a referral card with the name and number of a safe house run by the Women’s Rehabilitation Center (WOREC) in Nepal which provides legal counselling and shelter for victims. When contacted at the follow-up time point, measures were taken to ensure the safety of the women. First, the telephone conversations were continued only if the women themselves answered the phone. If they were alone and felt comfortable talking to the research assistant, an invitation was extended to attend a personal interview at the hospital. Appointments were made for the interviews, which were conducted with only the woman and research assistant present. 

### 2.2. Baseline Data Collection

Women agreeing to participate completed a Color-Coded Audio Computer-Assisted Self Interview (C-ACASI) on a tablet computer in the presence and, if necessary, with the help of a research assistant. C-ACASI allowed the participants to hear the interview questions through a headset as well as read and answer them on the tablet. We chose this mode of data collection in order to include women with limited literacy in the study. Except for the participants’ phone numbers, recorded confidentially for later follow-up (upon additional consent), all data were transferred electronically from the tablet to a safe server. Prior programming ensured a high-quality file for analysis. We used the most consistently used screening tool for intimate partner violence, which has been found to yield valid and specific identification of abuse when compared with longer abuse research instruments [26,27]. The Abuse Assessment Screen (AAS) consists of five items and was developed to detect abuse perpetrated against pregnant women [26,27]. The screening tool has been tested predominantly with young and poor women. We used the original AAS questions but changed the order and started with “Are you afraid of anyone in the family?” [25,26,27]. As in the original version, a positive answer to any question denotes abuse [26,27]. Women could not only answer yes or no, as in the original AAS, but also specify whom they were afraid of. We modified the Abuse Assessment Screen (AAS) by removing the body-map used to register the location of injuries sustained by a women experiencing violence [27]. The interview also included questions on socio-demographics and obstetric history and the five-item Hopkins Symptoms Checklist (HSCL-5), which had been used previously in a Nepali setting, to measure the emotional well-being of the women [28]. 

### 2.3. The safety Measures Checklist

Participants reporting DV were asked if they had practiced any safety measures. The safety measures assessed in our study were translated and adapted from the safety-promoting behavior checklist (SBC) used in a study among pregnant women by McFarlane et al. [15]. To ensure the quality and relevance of the safety measures in the Nepali context, we did forward-and-backward translations of the text in the checklist. We also adapted and modified the fifteen safety measures to the context (Table S1). Women in Nepal could not apply three of the safety measures in the original checklist because such systems are not in place in the country; namely, having available a social security number, receipts from rent and utility bills, and insurance policies and numbers. These items were replaced with three related but new safety measures that could be relevant for Nepali women: having available a citizenship card, the address of a safe house/shelter, and an extra sim card for a mobile phone. In addition, the items in the original checklist were elaborated and exemplified to make them context-specific for the women. For example, for the item described as “removing weapons”, the interviewer explained to participants that this could refer to hiding knives or guns in a haystack or a cupboard.

### 2.4. Follow-up Data Collection

Of the 1010 women who participated in the study at baseline, 181 reported experiences of DV. One of the co-authors was able to contact 80 of the 181 eligible women postpartum, and 62 agreed to the follow-up interview. In order not to raise the partner’s or family’s suspicion about the true reason for the visit and to inconvenience women as little as possible, appointments for the interviews were scheduled as much as possible to coincide with the women visiting the hospital for other reasons. However, for 29 women, a special hospital visit was scheduled for the follow-up research. One of the co-authors conducted the follow-up interviews in a private room using the same C-ACASI method as at baseline. The questionnaire at follow-up included questions on DV, safety measures practiced, and emotional well-being, using the same instruments as at baseline. In addition, at follow-up, women were asked about their living conditions after the earthquakes and how they felt about safety measures being taught to pregnant women. The interviews took place between 6 months and 21 months postpartum. All women were offered travel allowances for the follow-up interviews. 

### 2.5. The Educational Intervention

Our intervention consisted of teaching women 15 safety measures using a flip chart of text and illustrations (available on request). The duration of the teaching session lasted for a maximum of 30 min. Prior to the intervention, the first author discussed each safety measure in detail with a local artist to ensure context-sensitive illustrations were made. The flipchart consisted of participants’ pages, with the illustrations for the women, and teachers’ pages, with instructions for the education session. The instructions consisted of short summary points of information. For example, if the women were taught to hide money, the text for the teachers included suggestions on where women could hide money (in a box of clothes, inside a sack of rice, saved in a bank account). These served as potential prompts for the teachers. In the study, the flip chart was delivered by research assistants who were recruited and trained by the first author, who had extensive previous experience in this field. 

A preliminary version of the flipchart was pretested among a heterogeneous group of women that consisted of medical doctors, lecturers, medical students, nurses, cleaners, ward attendants, administrative staff at KMC, and the research team. The group was asked if they understood the drawings and the text and was invited to comment on the intervention as a whole. Their comments were used to adjust the flipchart. For example, the test group felt that the dress the women were wearing in the initial illustrations only represented rural women. This was changed to ensure representativeness of women from urban areas as well. 

### 2.6. Variables

#### 2.6.1. Safety Measures Variables

The safety behavior checklist measures the use of the behaviors taught in the intervention (Table S1), prior to the intervention and postpartum [15]. Women were asked “When you experienced violence, have you ever used any of the following safety measures?” Answering options were “yes”, “no”, or “not applicable”. For each woman, the total reported count of safety measures used was the sum score of safety measures, both at baseline and follow-up. The minimum score was thus 0, and maximum score 15. 

#### 2.6.2. Domestic Violence Variables

An affirmative response to any of the five AAS screening questions for DV was classified as having reported “any form of DV”, and those with all negative responses were classified as having “no exposure”. Sub-categories were created within the group of women who had reported of any DV. Women who reported they feared someone in the family but gave negative responses to any other experience of DV were classified as having reported “fear only”. Women who responded affirmatively to any physical, emotional, or sexual violence, but not to fear, were classified as being exposed to “violence only”. Women with affirmative responses to the questions on fear and violence were classified as having reported “both fear and violence”. Domestic violence was computed in the same four categories as in our previous publication [25]. 

#### 2.6.3. Other Variables

Women who reported having their own income were asked if they could decide how to use it. The women were then divided into three groups: “no income”; “income, no autonomy”; and “income and autonomy.” Income without autonomy indicates that a woman hands over her income to the husband/family without being able to decide how it is spent. Having autonomy denotes a woman can keep either some or all to spend at her own discretion. The women were categorized as “nulliparous” if they had never given birth and “multiparous” if they had children and/or a history of stillbirth. Gestational age indicating how far the pregnancy had come by the time of baseline assessment was self-reported. Women were categorized into four groups of caste/ethnicity: Dalits and religious minorities; disadvantaged Janajati and the disadvantaged group from the Terai region; advantaged Janajati; and upper caste. Women were considered to have symptoms of either anxiety or depression if their HSCL-5 score was more than two, at both baseline and follow-up [29]. 

### 2.7. Statistical Analysis

Statistical analyses were performed with SPSS version 22 (Statistical Product and Service Solutions) IBM, New York, USA. Missing data were not replaced. We performed detailed descriptive and comparative analyses (Chi-squared tests), comparing women who reported DV at both baseline and follow-up to women who reported DV at baseline only. 

## 3. Results

Of 1010 participants assessed for DV at baseline during pregnancy, 181 women reported DV. It proved impossible to reach 91 (50.3%) of these women postpartum. Another 27 women were unable to participate in a follow-up interview, and one woman we contacted postpartum had been misclassified as reporting DV at baseline. The final sample at follow-up comprised 62 women. We did not find any significant differences in the characteristics of the women participating in the follow-up and those lost to follow-up (Table S2). There were no significant differences in the reported use of safety measures at baseline among women who were recruited into the study before and after the earthquakes (Table S3). For half of the women who participated at follow-up, the earthquakes had destroyed their houses (Table 1). At follow-up, slightly more than half of the women (51.5%, or *n* = 32) reported any form of DV and 48.3% (*n* = 30) did not report any DV. The women who reported DV both at baseline and follow-up were older than women reporting DV at baseline only; otherwise, there were no significant differences in the background characteristics between these groups (Table 1). 

### 3.1. Findings for the Women Reporting DV at Baseline Only

Of the 30 women who reported DV at baseline only, 11 of them reported violence only, 18 reported fear only, and one reported fear and violence (Table 2). Sixteen women reported their husband or boyfriend as the perpetrator of the DV or the person they feared (Table 2). 

Mothers- and fathers-in-law and other members of the family were feared, but not once were they reported as perpetrating physical or sexual violence (data not in tables). The number of safety measures used at follow-up increased from 36 to 46 for the women who reported DV only at baseline (Table 3). Of the women reporting DV at baseline only, 9 had an increase in the number of safety measures they used, 14 had no change, 7 had a decrease from baseline to follow-up (data not in tables). All women who had an increase in the number of safety measures also used new measures for the first time at follow-up. One woman did not increase the total number of safety measures but reported 3 new measures at follow-up. The range of safety measures used increased from 10 to 14 at follow-up.

### 3.2. Findings for the Women Reporting Violence at Baseline and Follow-Up

The proportion of women reporting fear and violence was higher at follow-up than at baseline (*p* = 0.010) (Table 2). Of the 32 women reporting DV at both time points, 15 (47%) reported the same type of DV at baseline and follow-up, 1 fear and violence only, and 14 fear only (data not in tables). More than half of the women (17 of 32) reported a different form of DV at follow-up from baseline (data not in tables). Seven women reported an increase from fear only at baseline to fear and violence at follow-up. One woman changed her report from fear only to violence only. Two women changed from fear and violence to violence only, and three from reporting violence only at baseline to violence and fear at follow-up. Finally, four women changed their report from violence only to fear only. The proportion of women reporting their husband (or boyfriend or ex-husband) increased from 19 at baseline to 26 at follow-up (Table 2). 

Of the 32 women who reported DV at both assessment times, 10 women had an increase in the number of safety measures they used, 14 women had no change, and 8 had a decrease from baseline to follow-up (data not in tables). From baseline to follow-up, the total number of safety measures used increased from 56 to 80 and the number of not applicable decreased (Table 4). 

At follow-up, nineteen (60%) women reported using new safety measures on the list, measures that they had not used previously, at baseline (data not in tables). Four women with no change in the total number used new safety measures, while 4 women with a decrease in number reported also used new measures at follow-up. All women who had an increase in the number of safety measures reported using new measures at follow-up. The range of safety measures used increased from 7 to 11 at follow-up. There was no significant difference in family structure at follow-up between the women reporting violence at baseline only and the women reporting violence at baseline and follow-up (data not in tables).

## 4. Discussion

At follow-up, less than half of the women reported any form of DV. Significantly, more of the women who reported DV at follow-up reported the experience of both violence and fear at baseline, compared with those not reporting DV at follow-up. Women reporting DV at baseline and follow-up used more safety measures at baseline and follow-up compared with women reporting DV at baseline only. They also used more safety measures for the first time at follow-up. The range of safety measures used increased from baseline to follow-up both for women reporting DV at both assessments and for women reporting DV at baseline only. 

We had not expected that as many as half of the women we were able to contact would no longer report DV at follow-up. If true, this is a very positive finding. However, DV tends to be under-reported [30], but these women had previously disclosed DV. Women no longer reporting DV were not asked what they thought the reasons were for this change. One possible reason for DV to stop for a woman is when she removes herself from the situation(s) in which the DV takes place, for example by moving out from the joint family home or leaving the abusing partner. While the reason for leaving is to escape violence, there is evidence that the risk of harm actually increases around the time a woman leaves [31]. All women in this study received a card with contact details of a safe house. This may have encouraged some women to take actions. None of the safety measures explicitly advise women to leave their current home. However, several of the measures can be viewed as preparing women for the option of leaving. Even though half of the participants interviewed postpartum no longer reported DV, one third of them still reported using safety measures. One reason for this could be misclassification due to the wording of the question, “when you experienced violence, have you ever…”. This way of asking could lead women to report the same safety measure used previously once more at the second interview. However, this wording does not explain the new measures that women reported using. This suggests that women no longer reporting DV still find the safety measures useful. In addition, some measures are unlikely to be taken several times, such as making available a copy of a marriage certificate or other papers.

There was a difference between the women reporting DV at baseline only and those reporting DV at both times of contact. Of the women reporting DV twice, a substantially larger proportion reported both fear and violence at baseline. There also appeared to be an increase in the violence from baseline to follow-up for these women. Our results confirm that the measures are most useful for women experiencing DV, as this group of women had the largest increase in number of safety measures used from baseline to follow-up and the most new safety measures used for the first time at follow-up [32]. 

There are no “one size fits all” interventions that trigger the turning point or end of DV for pregnant women [33]. Women’s decision-making regarding the disclosure of DV and adoption of safety measures, or to leave violent relationships, is challenged by the complex realities and host of social and cultural factors that women face in abusive relationships. However, for decades, DV theorists have called attention to strategic moments, or “open window phases”, in the cyclical dynamics of DV—periods wherein women may be more open to help-seeking and interventions to increase their safety and well-being [34,35]. Evidence is increasingly suggesting that pregnancy is an open window phase for women and health care providers to address DV [36]. 

Safety measures are intended to promote the security and wellbeing of women living with abuse, help them prepare for an emergency, and protect themselves and their children [32]. Kennedy et al. also underscore the importance of effective formal help from interventions [37]. We do not know which safety measures women found most useful in our study nor if they experienced any barriers to use of safety measures, or had suggestions for other measures that should be included in a teaching session for women reporting DV. We also do not know if women in our study will be more likely to seek formal help in the future based on their experience of this intervention. Such questions would be prudent to explore in future intervention studies to account for the influence of various contextual factors and, importantly, ensure the intervention meets women’s actual needs.

The safety measures intervention was originally developed for English- and Spanish-speaking women experiencing ongoing intimate partner violence during pregnancy in the USA [15]. McFarlane et al. tested their intervention by delivering three teaching sessions during pregnancy and at three additional postpartum visits [15]. In contrast to McFarlane et al. [15], we included women with a diversity of types and severity of DV and had only one teaching session during pregnancy. McFarlane et al. presented a similar proportion of women using safety measures at baseline compared to our study, 48% vs. 50% respectively [15]. Our pilot study did not allow us to reproduce the significant increase in the use of safety measures from start to follow-up, which McFarlane showed [15]. However, in agreement with the pilot study of safety measures from Peru, we found an increase in the use of safety measures after intervention [18]. While a significant increase in the adoption of safety measures may be of some value, as the measures aim to protect women from more serious physical harm and aid her in being able to leave if needed, the best result for women is not requiring safety measures anymore due to the cessation of DV. 

This is the first study reporting the development and use of a flipchart to teach safety measures to pregnant women reporting DV in a routine antenatal care setting in Nepal. The flipchart was carefully and locally developed in order to capture the potential safety measures women may be able to use in this low-income setting. In our study, we used research assistants to assess DV and teach safety measures. In a routine ANC setting this would or should be done by health care providers. A qualitative study of public health midwives in Sri Lanka revealed that midwives acknowledged their unique status as trusted health providers in the communities and apply creative strategies (threaten abusive husbands, show images of babies born with deformities) to address DV among pregnant women [38]. 

## 5. Limitations of the study 

The unforeseen disruption of the earthquakes and consequent late follow-up of women in our study hampered the results. It contributed to a smaller sample than anticipated at the follow-up time point, limiting the analyses we could perform and conclusions we can draw from the study. Ideally, all women who reported DV at baseline should have been included in the follow-up, and we were only able to contact half of the women. Nevertheless, we found no significant differences between participants and non-participants at follow-up, indicating no selection bias on these characteristics at baseline. We cannot fully assess the effect of the earthquakes on our study. Nevertheless, we found no significant difference in the use of safety measure nor the proportion of women reporting DV before and after the earthquakes. 

Our study did not include a comparison group. The optimal design for testing an intervention is a randomized controlled trial, as was done by McFarlane et al. [32]. We considered conducting an Randomized Controlled Trial (RCT) but received expert advice that such a design was premature in our setting, where the prevalence of DV during pregnancy was unknown and the intervention had to be developed.

## 6. Conclusions

Our study provides a description of the development of a flipchart education intervention promoting the use of safety measures during routine ANC for pregnant women who have reported DV in Nepal. There was an increase in the adoption of safety measures after intervention. Our study is a pilot for others planning larger studies including control groups in low-income settings. Further qualitative studies assessing the relevance and use or non-use of safety measures following the intervention would be beneficial. In addition, it would be useful to assess the benefit of teaching the safety measures repeatedly, in particular to women experiencing severe violence. A process evaluation would be valuable to understand the contextual factors that affect pregnant women’s use of safety measures.

## Figures and Tables

**Table 1:** Descriptive characteristics at baseline of women reporting Domestic Violence (DV), *N* = 62.

		Reporting DV at Baseline Only *n* = 30	Reporting DV at Baseline and Follow-Up *n* = 32	Total *n* = 62	*p*-Value
		*n* (%)	*n* (%)	*n* (%)	
Women’s characteristics					
Age in years	15–19	4 (13.3)	0.0	4 (6.5)	0.02
	20–24	19 (63.3)	14 (43.8)	33 (53.2)	
	25–29	3 (10.0)	10 (31.2)	13 (21.0)	
	≥30	4 (13.3)	8 (25.0)	12 (19.4)	
Education	None	3 (10.0)	5 (15.6)	8 (12.9)	0.46
	Primary	7 (23.3)	3 (9.4)	10 (16.1)	
	Secondary	7 (23.3)	10 (31.2)	17 (27.4)	
	Higher secondary and above	13 (43.3)	14 (43.8)	27 (43.5)	
Income	No income	22 (73.3)	25 (78.1)	47 (75.8)	0.72
	Income no autonomy	1 (3.3)	2 (6.2)	3 (4.8)	
	Income and autonomy	7 (23.3)	5 (15.6)	12 (19.4)	
Parity	Nulliparous	12 (40.0)	12 (37.5)	24 (38.7)	1.00
	Multiparous	18 (60.0)	20 (62.5)	38 (61.3)	
Gestational age in weeks	Mean ± SD	21.7 ± 4.1	21.09 ± 4.8	21.4 ± 4.4	
Baseline anxiety and depression	≤2 score	9 (30.0)	7 (21.9)	16 (25.8)	0.66
	>2 score	21 (70.0)	25 (78.1)	46 (74.2)	
Husband’s characteristics					
Age in years	≤24	4 (14.3)	2 (6.5)	6 (10.2)	0.30
	25–29	8 (28.6)	13 (41.9)	21 (35.6)	
	30–34	11 (39.3)	7 (22.6)	18 (30.5)	
	≥35	5 (17.9)	9 (29.0)	14 (23.7)	
Education	None	4 (13.8)	3 (9.7)	7 (11.7)	0.62
	Primary	5 (17.2)	3 (9.7)	8 (13.3)	
	Secondary	6 (20.7)	11 (35.5)	17 (28.3)	
	Higher secondary and above	14 (48.3)	14 (45.2)	28 (46.7)	
Family/community characteristics					
Family structure	Nuclear	15 (51.7)	21 (75.0)	36 (63.2)	0.10
	Extended	14 (48.3)	7 (25.0)	21 (36.8)	
Geographical setting	Rural	6 (20.0)	3 (9.4)	9 (14.5)	0.34
	Urban	24 (80.0)	29 (90.6)	53 (85.5)	
Caste/Ethnicity	Dalit	0.0	0.0	0.0	0.71
	Disadvantaged Janajati	10 (38.5)	8 (29.6)	18 (34.0)	
	Advantaged Janajati	3 (11.5)	5 (18.5)	8 (15.1)	
	Upper caste	13 (50.0)	14 (51.9)	27 (50.9)	
House destroyed by earthquake	Yes	13 (43.3)	19 (59.4)	32 (51.6)	0.31
	No	17 (56.7)	13 (40.6)	30 (48.4)	

**Table 2:** Type of DV and number of safety measures reported, *N* = 62.

	Women Reporting DV at Baseline Only *n* = 30		Women Reporting DV Both Times *n* = 32	
	**Baseline**		**Baseline**	**Follow-up**
	*n* (%)		*n* (%)	*n* (%)
Type of DV reported *				
Fear only	18 (60.0)		22 (68.8)	18 (56.3)
Violence only	11 (36.7)		3 (9.4)	3 (9.4)
Fear and violence	1 (3.3)		7 (21.9)	11 (34.4)
Perpetrator or person feared **				
Husband or boyfriend	16 (53.3)		19 (59.3)	26 (80.1)
Mother- or father-in-law, other family	10 (33.3)		10 (31.2)	7 (21.9)
None registered	7 (23.3)		8 (25.0)	4 (12.5)
Frequency of DV (including fear) reported				
Most of the time/often ***	11 (36.6)		13 (40.6)	14 (43.7)
Sometimes/seldom	14 (46.6)		16 (50.0)	17 (53.1)
Not reported	5 (16.6)		3 (9.4)	2 (6.2)
Number of behaviors used				
	Baseline	Follow-up	Baseline	Follow-up
None	19	20	12	14
One	4	2	7	4
Two	2	3	3	5
Three	2	1	4	1
Four	0	1	2	0
Five	0	1	2	1
Six	2	0	1	1
Seven	0	0	1	1
Eight	0	0	0	2
Nine	0	0	0	2
Ten	1	0	0	0
Eleven	0	0	0	1
Twelve	0	1	0	0
Fourteen	0	1	0	0

* Difference at baseline Chi-squared *p*-value = 0.010, ** more than 100% possible, *** includes women reporting several episodes during last year.

**Table 3:** Safety measures practiced at recruitment by women who reported DV at baseline only, *n* = 30.

	At Baseline	Not Applicable at Baseline	At Follow-Up	Not Applicable at Follow-Up	New Practices at Follow-up	New not Applicable at Follow-Up
Safety Measures	*n*	*n*	*n*	*n*	*n*	*n*
Had ever:						
Hid money	0	6	3	6	3	3
Hid important keys	1	4	2	6	1	5
Established codes with family and friends	2	5	4	6	3	4
Asked neighbors to call the police	1	7	4	10	3	5
Removed weapons	2	8	2	9	2	4
Had made available:						
Marriage certificates	6	6	5	7	2	3
Birth certificates (yours and your child)	4	7	6	7	4	2
Bank account numbers	2	5	2	9	1	6
Citizenship card	4	4	4	7	3	4
Driver’s license	0	10	1	8	1	3
Valuable jewelry with bills	3	5	4	8	2	4
Important phone number/s	3	6	4	8	1	4
Hidden bag with extra clothing	2	6	2	8	1	4
Address for safe house/shelter	4	8	2	7	1	1
Extra sim card	2	7	1	8	0	3
Total number of safety measures	36	94	46	114	28	55

**Table 4:** Safety measures practiced at baseline and follow-up and for the first time at follow-up for women reporting DV both at baseline and at follow-up, *n* = 32.

	At Baseline	Not Applicable at Baseline	At Follow-Up	Not Applicable at Follow-Up	New at Follow-Up	New not Applicable at Follow-Up
	*n*	*n*	*n*	*n*	*n*	*n*
Had ever:						
Hid money	2	7	5	7	5	4
Hid important keys	2	5	2	7	2	4
Established codes with family and friends	6	8	6	6	4	4
Asked neighbors to call the police	3	7	4	4	4	1
Removed weapons	1	9	2	4	2	1
Had made available:						
Marriage certificates	13	7	10	3	2	2
Birth certificates (yours and your child)	7	8	9	4	7	1
Bank account numbers	3	7	5	5	4	2
Citizenship card	10	7	10	2	4	0
Driver’s license	1	8	2	4	2	2
Valuable jewelry with bills	0	10	7	5	7	1
Important phone number/s	4	10	5	6	4	2
Hidden bag with extra clothing	2	10	7	4	5	1
Address for safe house/shelter	1	9	5	6	4	1
Extra sim card	1	9	1	5	1	1
Total number of safety measures	56	121	80	72	57	27

## Data Availability

The datasets generated and/or analyzed during the current study are not publicly available due to the need to restrict access to known persons only but are available from the corresponding author on reasonable request.

## Supplementary Materials

The following are available online at https://www.mdpi.com/1660-4601/17/7/2268/s1, Table S1: Original and modified safety-promoting behavior checklist, Table S2: Characteristics at baseline for women who participated in follow-up and those lost to follow-up, *N* = 181, Table S3: Safety measures practiced * at baseline by women who reported DV, *N* = 181.
