# Supplementary material for: Improving Safety Among Pregnant Women Reporting Domestic Violence in Nepal—A Pilot Study
Source: Int J Environ Res Public Health. 2020 Mar 27;17(7):2268. doi: 10.3390/ijerph17072268 (PMC7177396; doi:10.3390/ijerph17072268)
Supplement: Supplementary file 1 [file ijerph-17-02268-s001.pdf]

Table S1. Original and modified safety-promoting behavior checklist.

Safety-Promoting Behavior Checklist<br>Ask the Women to Answer "Yes", "No" or "Not Applicable"				
**Original text**	**Modified text**	**Yes**	**No**	**Not applicable**
Have you ever	Have you ever			
Hid money	Hid money			
Hid an extra set of house and/or car keys	Hid important keys (important keys means house keys, car keys or any other vehicle keys)			
Established code with family or friends	Established code with family and friends (established code means made agreements with friends and family such as a hidden message when violence begins and you need help)			
Asked neighbors to call the police if violence begins	Asked neighbors to call the police if violence begins			
Removed weapons	Removed weapons (hidden or removed weapons like knives, guns, etc.)			
Have you ever made available	Have you ever made available			
Social security number (yours, his, children)	Birth certificates (your birth certificate and your children's birth certificates)			
Rent and utility receipts	Bank account numbers			
Birth certificates (yours and children)	Citizenship card			
ID or driver's license	Driver's license			
Bank account numbers	Marriage license/certificates			
Insurance policies and numbers	Valuable jewelry/ies and the bills			
Marriage license	Important phone number/s			
Valuable jewelry	Hidden bag with extra clothing			
Important phone numbers	Address for safe house/shelter			
Hidden bag with extra clothing	Extra mobile sim card			

Table S2. Characteristics at baseline for women who participated in follow-up and those lost to follow-up, $N = 181$.

Characteristics		Follow-up		
		Participants	Non-participants	
		$N = 62$<br>n (%)	$N = 119$<br>n (%)	p-value
Women's characteristics				
Age in years	15–19	4 (6.5)	9 (7.6)	0.145
	20–24	33 (53.2)	64 (53.8)	
	25–29	13 (21.0)	36 (30.3)	
	≥30	12 (19.4)	10 (8.4)	
Education	No	8 (12.9)	25 (21.0)	0.561
	Primary	10 (16.1)	19 (16.0)	
	Secondary	17 (27.4)	32 (26.9)	
	Higher secondary and above	27 (43.5)	43 (36.1)	
Income	No income	47 (75.8)	94 (79.0)	0.258
	Income no autonomy	3 (4.8)	11 (9.2)	
	Income and autonomy	12 (19.4)	14 (11.8)	
Parity	Nulliparous	24 (38.7)	61 (51.3)	0.073
	Multiparous	38 (61.3)	58 (48.7)	
Gestational weeks at baseline in weeks	Mean ± SD	21.40±4.42	20.64±11.37	
Husband's characteristics				
Age in years	≤24	6 (10.2)	30 (26.3)	0.025
	25–29	21 (35.6)	45 (39.5)	
	30–34	18 (30.5)	25 (21.9)	
	≥35	14 (23.7)	14 (12.3)	
Education	No	7 (11.7)	12 (10.5)	0.838
	Primary	8 (13.3)	21 (18.4)	
	Secondary	17 (28.3)	33 (28.9)	
	Higher secondary and above	28 (46.7)	48 (42.1)	
Family/community characteristics				
Family structure	Nuclear	36 (63.2)	58 (51.8)	0.191
	Extended	21 (36.8)	54 (48.2)	
Geographical setting	Rural	9 (14.5)	7 (5.9)	0.094
	Urban	53 (85.5)	112 (94.1)	
Caste/ethnicity	Dalit		3 (3.7)	
	Disadvantaged Janajati	18 (34.0)	14 (17.3)	0.086
	Advantaged Janajati	8 (15.1)	17 (21.0)	
	Upper caste	27 (50.9)	47 (58.0)	
Baseline anxiety and depression	≤2 score	16 (25.8)	39 (32.8)	0.396
	>2 score	46 (74.2)	80 (67.2)	
Reported violence at baseline	Fear only	40 (64.5)	69 (58.0)	0.155
	Violence only	14 (22.6)	21 (17.6)	
	Both violence and fear	8 (12.9)	26 (24.4)	
Earthquake status	Before	28 (45.2)	66 (55.5)	0.212
	After	34 (54.8)	53 (44.5)	

Table S3. Safety measures practiced* at baseline by women who reported DV, *N* = 181.

	**Before Earthquake *N* = 94**	**After Earthquake *N* = 87**
**Safety measures**	**n (%)**	**n (%)**
Had ever:		
Hid money	3 (3.2)	5 (5.7)
Hid important keys	3 (3.2)	6 (6.9)
Established codes with family and friends	10 (10.6)	15 (17.2)
Asked neighbors to call the police	4 (4.3)	8 (9.2)
Removed weapons	5 (5.3)	1 (1.1)
Had made available:		
Birth certificates (yours and your child)	16 (17.0)	15 (17.2)
Bank account numbers	9 (9.6)	9 (10.3)
Citizenship card	18 (19.1)	23 (26.4)
Driver's license	3 (3.2)	2 (2.3)
Marriage certificates	19 (20.2)	24 (27.6)
Valuable jewelry with bills	10 (10.6)	11 (12.6)
Important phone number/s	9 (9.6)	14 (16.1)
Hidden bag with extra clothing	12 (12.8)	8 (9.2)
Address for safe house/shelter	15 (16.0)	18 (20.7)
Extra sim card	7 (7.4)	7 (8.0)
Number of women using any safety measure	50 (53.2)	52 (59.8)

*More than one safety measure could be used by an individual woman, resulting in more than 100% number of measures.
